# Describing a Persistent SARS‐CoV‐2 Infection Outcomes and Mutations Associated With B‐Cell Deficiency: A Case Report

**DOI:** 10.1155/crdi/6154094

**Published:** 2026-09-24

**Authors:** Rana Mohamed, Alex Shipe, Andrew Lail, Isla Emmen, William Vuyk, Nicholas Minor, Taylor Bradley, Angela Gifford, Nancy Wilson, David H. O’Connor, Jacqueline Garonzik-Wang, Jeannina Smith

**Affiliations:** ^1^ Department of Surgery, University of Wisconsin-Madison, 600 Highland Ave, Madison 53792, Wisconsin, USA, wisc.edu; ^2^ Department of Pathology and Laboratory Medicine, University of Wisconsin-Madison, 5510 Element Way, Madison 53719, Wisconsin, USA, wisc.edu; ^3^ Department of Surgery, University of Alabama at Birmingham, 1808 7th Ave South Boshell Diabetes Building #505, Birmingham 35233, Alabama, USA, uab.edu; ^4^ Department of Medicine, University of Wisconsin-Madison, 600 Highland Ave, Madison 53792, Wisconsin, USA, wisc.edu

**Keywords:** immunocompromised patients, persistent infections, SARS-CoV-2, viral lineage, viral mutations

## Abstract

**Background:**

Immunocompromised (IC) individuals are at an increased risk for persistent SARS‐CoV‐2 infections and can develop new viral mutations and lineages not seen in the community. In this case report, a persistent SARS‐CoV‐2 infection (330 days) in an IC patient is examined for viral mutations and mutations associated with cryptic lineages.

**Case Presentation:**

The patient was followed up in a longitudinal study examining persistent SARS‐CoV‐2 in IC patients. The patient provided stool and nasal swab samples biweekly until 28 days postenrollment, then monthly, and then quarterly after 12 months post enrollment until the participant was no longer positive for SARS‐CoV‐2. Staff performed RT‐qPCR and viral sequencing on the samples. Viral mutations from the XBK lineage were already present in the initial sample. By the end of the infection period, there were 40 fixed consensus changes from XBK of which two mutations were typical for cryptic lineages. Mutations increased steadily over time, with most mutations fixed by day 253, including the cryptic typical mutations.

**Conclusion:**

This case demonstrates the potential for persistent SARS‐CoV‐2 infections to develop mutations and lineages in IC patients and highlights the need for continued SARS‐CoV‐2 monitoring and treatment in this vulnerable population.

## 1. Introduction

Immunocompromised (IC) individuals living with B‐cell deficiencies have a greater susceptibility to prolonged infections due to their impaired ability to develop antibodies [1, 2]. This risk of infection persistence is of particular concern for SARS‐CoV‐2 infections, as IC patients are at a higher risk for severe SARS‐CoV‐2 infection [3–5] and can become persistent viral shedders leading to increased viral spread [6]. We examined persistent infection in 40 adult IC patients: 39 solid organ transplant (SOT) recipients and one individual with a B‐cell deficiency. The data from the SOT cohort were previously published in Vuyk et al. [7]. Of 30 SOT recipients, 12 had positive SCV2 RNA at 28 days or more after testing positive; one subject had continual detection out to 54 days after the first positive test. The current report describes the persistent infection and SARS‐CoV‐2 mutations of the B‐cell‐deficient IC patient who had continued detection of SARS‐CoV‐2 for 330 days. A preprint has previously been published by Mohamed et al. [8].

## 2. Case Presentation

### 2.1. Study Timeline

The patient was identified and referred by the PI as a potential study candidate after testing positive for SARS‐CoV‐2 in December 2023 (d0 days post enrollment—dpe) and self‐reporting COVID‐19 symptomology for several months. This patient had mantle cell lymphoma in the past and was treated initially with chemotherapy followed by an autologous stem cell transplant. After 5 years, the mantle cell lymphoma relapsed, and they were treated with lymphodepletion chemotherapy followed by CD 19/20 CAR T infusion. The PI confirmed eligibility based on protocol‐specified criteria. Following referral, the research team contacted the patient remotely to discuss participation, and informed consent was obtained. The study was approved by the University of Wisconsin’s IRB.

### 2.2. Nasal Swab Viral Isolation and RT‐qPCR

Nasal swab samples were self‐collected, using nasal swab kits with Universal Transfer Medium (Fisher Scientific, 23600989) and shipped to the processing and storage laboratory overnight. Samples were stored and then batched with other nasal swab samples to isolate on the KingFisher using the Maxwell HT Viral TNA Kit, Custom for the KingFisher (Promega, AX2340). RT‐qPCR was performed by the Wisconsin National Primate Center Virology Core, as described in our previous manuscript [7]. Briefly, they used 300 μL of the nasal swab to perform ultra‐sensitive quantitative PCR (qPCR) for SARS‐CoV‐2, analyzing nucleocapsid (N1 and N2) as well as human RNAseP as an internal positive control.

### 2.3. Stool Sample Viral Isolation and RT‐qPCR

Stool samples were self‐collected using stool collection kits (Norgen, Thorold, Ontario, Canada). These kits preserve RNA in stool samples for up to 7 days at room temperature. Total nucleic acids (TNAs) were isolated from approximately 300 μg of each stool sample using the Promega RSC Fecal Microbiome DNA kit (Promega, Fitchburg, WI, Cat # AS1700). The isolated TNA was tested with qPCR for SARS‐CoV‐2 nucleocapsid N1 and N2 targets using the GoTaq Enviro Wastewater SARS‐CoV‐2 System (Promega, Fitchburg, WI, Cat# AM2100) on a LightCycler 96.

### 2.4. Sequencing

Once we determined that a sample was positive for SARS‐CoV‐2 and that we had sufficient material for sequencing (Ct < 30), we batched samples and used the QIASeq DIRECT SARS‐CoV‐2 kit (333891) with enhancer (333884) and Region Booster (333897, all Qiagen) to perform overlapping amplicon amplification. Samples were normalized and pooled and then sent to the UW Biotech Center to sequence on the NovaSeq X. Data were analyzed using the oneroof pipeline (github: https://github.com/nrminor/oneroof). Pango lineages were determined using NextClade.

**FIGURE 1:**
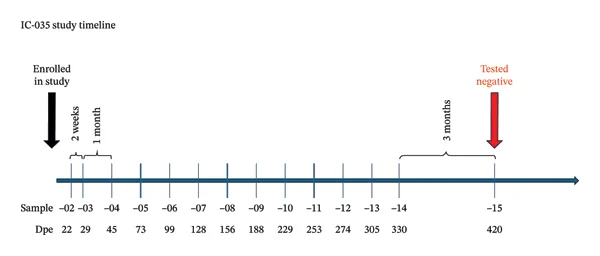
Timeline for study: participants were identified by study physicians and then enrolled by department of surgery clinical research personnel. Typically, a sample was obtained within 1 week of enrollment (sample‐01), then at Weeks 2 and 4, and monthly thereafter. After the first year on‐study, samples were obtained quarterly. The maximum enrollment was 2 years. For the current patient, the first sample was delayed longer than usual, so it became sample 02, and there was no sample‐01.

As shown in Figure 1, samples were obtained at day 14 (IC‐035‐02), day 28 (IC‐035‐03), and monthly thereafter. After the 1‐year anniversary, samples were collected quarterly to reduce participant burden. Once the participant tested negative for SARS‐CoV‐2 in both the nasal swab and stool samples, they were removed from the study. Our last samples from this participant were collected at time point IC‐035‐15, at which time both the stool and nasal swab samples tested negative. We have not included analysis of stool sample sequencing in this study because the quality of the sequence from stool was not sufficient.

### 2.5. Mutations From the Reference Baseline Isolate of XBK Were Already Present at the Earliest Time Point

**FIGURE 2:**
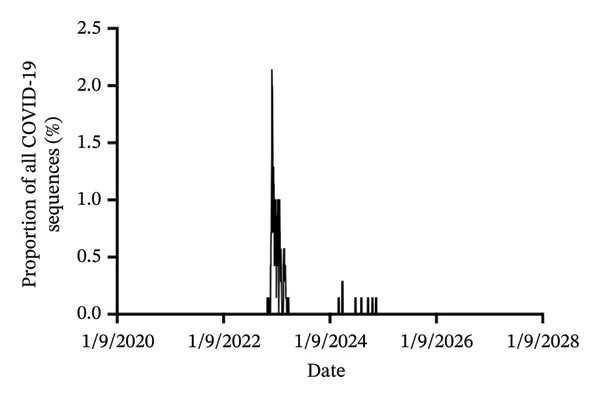
XBK was primarily in circulation between November 2022 and April 2023 [9]. The small spikes in 2024 are the patient’s samples.

Although the patient was enrolled in our study in early 2024, sequencing at the first time point revealed that the XBK lineage caused the infection. XBK is a recombinant between BA.5.2 and CJ.1, first isolated in Denmark in October 2022. As no GenBank accession is specified in the original Pango designation for XBK, we used the earliest available GenBank genome assigned to lineage XBK (OY837126; Denmark, collected 14 Oct 2022) as a representative reference sequence for comparative analyses. The peak of circulation for this virus was between November 2022 and April 2023 (Figure 2). Therefore, it is likely that the patient was infected between 8 months and 1 year before enrollment into the study. In all, 472 sequences have been uploaded to GenBank, of which 82 were from the USA, 47 were collected in WI, and 34 were from this participant.

At the earliest time point, 22 dpe, there were already eight consensus mutations when comparing to the baseline reference XBK sequence (Table 1, Table 2), potentially reflecting ongoing mutation between the time the participant was infected and the first samples. We compared 19 other XBK sequences from the USA that were the most recent entries in GenBank, excluding our IC‐035 uploaded sequences to IC‐035‐NS. We found only one sequence that was closer to our earliest sequence, with 6 mutational differences, a sequence from IL on 1/28/23, nearly a year before. All of the other sequences were more disparate from IC‐035‐02 (Supporting Table 1). Therefore, we felt comfortable using the baseline reference, as we did not appear to be biasing the data.

**TABLE 1:** Changes found in the original XBK sequence (OY837126) compared to Wuhan‐1^a^.

XBK	
Canonical mutation	Canonical mutation
orf1ab:p.Ser135Arg	ORF1a:S135R
orf1ab:p.Thr842Ile	ORF1a:T842I
orf1ab:p.Ser1221Leu	ORF1a:S1221L
orf1ab:p.Gly1307Ser	ORF1a:G1307S
orf1ab:p.Pro1640Ser	ORF1a:P1640S
orf1ab:p.Leu3027Phe	ORF1a:T3090I
orf1ab:p.Thr3090Ile	ORF1a:L3201F
orf1ab:p.Leu3201Phe	ORF1a:T3255I
orf1ab:p.Thr3255Ile	ORF1a:P3395H
orf1ab:p.Pro3395His	ORF1a:del3675/3677
orf1ab:p.Asn4060Ser	ORF1a:N4060S
orf1ab:p.Pro4715Leu	ORF1b:P314L
orf1ab:p.Gly5063Ser	ORF1b:G662S
orf1ab:p.Arg5716Cys	ORF1b:R1315C
orf1ab:p.Ile5967Val	ORF1b:I1566V
orf1ab:p.Val6107Ile	ORF1b:V1706I
orf1ab:p.Thr6564Ile	ORF1b:T2163I
S:p.Thr19Ile	S:T19I
S:p.Ala27Ser	S:A27S
S:p.Gly142Asp	S:G142D
S:p.Lys147Glu	S:K147E
S:p.Trp152Arg	S:W152R
S:p.Phe157Leu	S:F157L
S:p.Ile210Val	S:I210V
S:p.Val213Gly	S:V213G
S:p.Gly257Ser	S:G257S
S:p.Gly339His	S:G339H
S:p.Arg346Thr	S:R346T
S:p.Ser371Phe	S:S371F
S:p.Ser373Pro	S:S373P
S:p.Ser375Phe	S:S375F
S:p.Thr376Ala	S:T376A
S:p.Asp405Asn	S:D405N
S:p.Arg408Ser	S:R408S
S:p.Lys417Asn	S:K417N
S:p.Asn440Lys	S:N440K
S:p.Gly446Ser	S:G446S
S:p.Asn460Lys	S:N460K
S:p.Ser477Asn	S:S477N
S:p.Thr478Lys	S:T478K
S:p.Glu484Ala	S:E484A
S:p.Phe483Pro	S:F486P
S:p.Phe490Ser	S:F490S
S:p.Gln498Arg	S:Q498R
S:p.Asn501Tyr	S:N501Y
S:p.Tyr505His	S:Y505H
S:p.Asp614Gly	S:D614G
S:p.His655Tyr	S:H655Y
S:p.Asn679Lys	S:N679K
S:p.Pro681His	S:P681H
S:p.Asn764Lys	S:N764K
S:p.Asp796Tyr	S:D796Y
S:p.Gln954His	S:Q954H
S:p.Asn969Lys	S:N969K
ORF3a:p.Thr223Ile	ORF3a:T223I
E:p.Thr9Ile	E:T9I
E:p.Thr11Ala	E:T11A
M:p.Gln19Glu	M:Q19E
M:p.Ala63Thr	M:A63T
ORF6:p.Asp61Leu	ORF6:D61L
N:p.Pro13Leu	N:P13L
N:p.Arg203Lys	N:R203K
N:p.Gly204Arg	N:G204R
N:p.Ser413Arg	N:S413R
ORF9b:p.Pro10Ser	ORF9b:P10S

^a^The reference baseline isolate of the XBK lineage had 65 mutations from Wuhan‐1. When analyzing consensus mutations over time, these mutations were removed so that we could determine which mutations were due to changes in XBK attributable to evolution while the patient was infected.

**TABLE 2:** Consensus mutations over time^a^.

			Time point	IC‐35‐02_NS	IC‐035‐03_NS	IC‐035‐04_NS	IC‐035‐05_NS	IC‐035‐06_NS	IC‐035‐07_NS	IC‐035‐08_NS	IC‐035‐09_NS	IC‐035‐10_NS	IC‐035‐11_NS	IC‐035‐12_NS	IC‐035‐13_NS	IC‐035‐14_NS
Pos	Mutation	Mutation	dpe	22	29	45	73	99	128	156	186	229	253	274	305	330
413	orf1ab:p.Thr50Pro	ORF1ab:T50P											63.6364			
520	orf1ab:p.Met85Ile	ORF1ab:M85I													55.5555	
3042	orf1ab:p.Pro926His	ORF1ab:P926H		99.8007	99.7958	99.8925	99.8731	99.7717	99.8972	99.409	99.7843	99.7262	99.6316	99.7873	99.7915	99.8572
3614	orf1ab:p.Val1117Ile	ORF1ab:V1117I										59.1685	99.8483	99.7818	99.8349	99.6625
4232	orf1ab:p.Asp1323Asn	ORF1ab:D1323N										59.5376	99.1597	99.8155	99.6516	100
4379	orf1ab:p.Ser1372Ala	ORF1ab:S1372A										53.4055	100	99.7435	99.9374	99.7063
6609	orf1ab:p.Arg2115Ile	ORF1ab:R2115I			88.0919		62.9747	89.7306		85.0062						
7798	orf1ab:p.Lys2511Asn	ORF1ab:K2511N										76.7554	99.8082	99.8672	99.8583	100
8354	orf1ab:p.Arg2697Cys	ORF1ab:R2697C											58.9818			62.8953
8394	orf1ab:p.Ala2710Val	ORF1ab:A2710V										79.0441	99.7499	99.7416	99.7263	99.6683
9042	orf1ab:p.Ser2926Phe	ORF1ab:S2926F							77.5547		99.551	99.604	100	99.691	99.9014	100
9711	orf1ab:p.Ser3149Phe	ORF1ab:S3149F							74.7998		94.3035	99.7152	99.7357	99.7137	99.6914	99.5321
9756	orf1ab:p.Arg3164His	ORF1ab:R3164H		97.2452	74.3663	53.357	91.786	95.5436	83.4333	92.4067	94.9883	99.5449	99.831	99.7813	99.7916	99.6568
11296	orf1ab:p.Phe3677Leu	ORF1ab:F3677L		93.47824	76.1905		77.7777	96.4286	86.66663	89.2858	97.7208	100	100	100.00004	100	100.00002
11669	orf1ab:p.Arg3802Cys	ORF1abR3802C										54.2229	99.8875	99.693	99.7283	100
12008	orf1ab:p.Leu3915Phe	ORF1ab:L3915F							67.2679							
12045	orf1ab:p.Ile3927Thr	ORF1ab:I3927T								74.0105						
12194	orf1ab:p.Leu3977Ile	ORF1ab:L3977I									86.7123	99.9356	99.87	99.9325	99.8787	100
12651	orf1ab:p.Thr4129Ile	ORF1ab:T4129I							69.97							
14584	orf1ab:p.Ala4774Thr	ORF1ab:A4774T									79.1887					
15181	orf1ab:p.Ala4973Thr	ORF1ab:A4973T									76.0095					
18985	orf1ab:p.Val6241Ile	ORF1ab:V6241I													53.4095	
19969	orf1ab:p.Ala6569Thr	ORF1ab:A6569T														63.2327
20404	orf1ab:p.Pro6714Ser	ORF1ab:P6714S										73.9298	99.7037	99.7532	99.7188	99.756
21137	orf1ab:p.Lys6958Arg	ORF1ab:K6958R								73.0872		74.1822	99.7506	99.7513	99.8201	99.9533
21160	orf1ab:p.Ala6966Thr	ORF1ab:A6966T														97.1429
21587	S:p.Pro9Ser	S:P9S													58.371	
21593	S:p.Val11Ile	S:V11I													58.371	
21641	S:p.Ala27Pro	S:A27P		80												
21718	S:p.Gln52His	S:Q52H			56.2609			76.9352		65.2367	75.9161	98.0392	98.951	99.1079	99.0911	100
21746	S:p.Val62Leu	S:V62L						74.247		74.2334		74.2459	100	100	99.8463	99.6479
21771	S:p.Val70Ala	S:V70A										74.2459	100	99.8788	99.7949	100
22295	S:p.His245Tyr	S:H245Y									82.7692	99.7741				
22296	S:p.His245Leu	S:H245L												61.5385		
22329	S:p.Ser256Leu	S:S256L										62.1289	99.8386	99.8314	99.9109	100
22629	S:p.Lys356Thr	S:K356T		82.2605	59.2885		87.5899	92.9348	78.4461	93.8556		70.5925	100	99.9369	100	100
22893	S:p.Lys444Thr	S:K444T			62.0667		58.3261	85.5016		78.3554		77.5588	99.9279	99.8583	99.8592	100
22925	S:p.Leu455Met	S:L455M							71.6089							
22993	S:p.Ser477Arg	S:S477R										71.9497	99.4866	99.5102	99.6241	99.3377
23013	S:p.Glu484Val	S:E484V														50.463
23014	S:p.Glu484Asp	S:E484D									56.1253					
23018	S:p.Phe486Pro	S:F486P		99.9452	99.878	99.7374	99.8715	99.9122	99.8848	99.8395	99.9323	99.964	99.966	99.9526	99.9674	97.5309
23069	S:p.Val503Leu	S:V503L			62.8467		58.138	85.923		79.8627		88.8621	99.5468	99.9088	99.8777	100
23099	S:p.Leu513Phe										71.6075					
23108	S:p.Glu516Gln	S:E516N		77.8598		82.27	85	83.4036	91.2486	73.1797						
23277	S:p.Thr572Ile	S:T572I										67.0603	99.195	99.7428	99.8739	99.8496
23453	S:p.Pro631Ser	S:P631S							62.6779							
23625	S:p.Ala688Val	S:A688V										84.3033	99.897	99.8255	99.8538	100
23954	S:p.Gly798Ser	S:G798S													56.3348	
24090	S:p.Asp843Gly	S:D843G								50.3644						
24423	S:p.Gln954Arg	S:Q954R							79.1635							
24441	S:p.Asn960Thr	S:N960T			75.6959		61.7587	88.7976		72.3933	66.9774	99.8292	100	99.9381	99.9535	
24453	S:p.Lys964Ile	S:K964I												57.2644	99.9536	
24863	S:p.His1101Tyr	S:H1101Y										67.5359	99.9629	99.8562	99.8649	100
25019	S:p.Asp1153Tyr	S:D1153Y													55.8231	56.5483
25496	ORF3a:p.Ile35Thr	ORF3a:I35T									72.3917					
25549	ORF3a:p.Leu53Phe	ORF3a:L53F										67.135	99.6559	99.6024	99.6693	100
25552	ORF3a:p.Ala54Ser	ORF3a:A54S										67.2702	99.9485	99.8063	99.802	100
26416	E:p.Val58Leu	E:V58L									68.5083					
26530	M:p.Asp3Gly	M:D3G														52.1411
26533	M:p.Ser4Phe	M:S4F		82.5127	62.5	79.5587	88.9081	92.3475	87.2399	76.1317		69.2817	99.8243	99.9185	99.5569	99.7481
26538	M:p.Gly6Ser	M:G6F									69.3009					
27632	ORF7a:p.Arg80Ile	ORF7a:R80I														58.6207
28365	N:p.Glu31Val	N:E31V												95.2381		
28367	N:p.Arg32Cys	N:R32C						84.6154								
28370	N:p.Ser33Gly	N:S33G					60.8696	58.3333			69.1176	66.6667	50.1	76.2963	61.165	78.9474
28610	N:p.Leu113Ile	N:L113I									90.9051	99.8044	99.8135	99.7966	99.7741	99.9477
28821	N:p.Ser183Phe	N:S183F														100
29663	ORF10:p.Asn36Tyr	ORF10:N36Y										82.4596	99.8378	99.708	99.8358	99.7344

^a^This table shows the frequencies of mutations that were observed in nasal swab sequences from this patient over time.

### 2.6. Increasing Mutations Over Time

**FIGURE 3:**
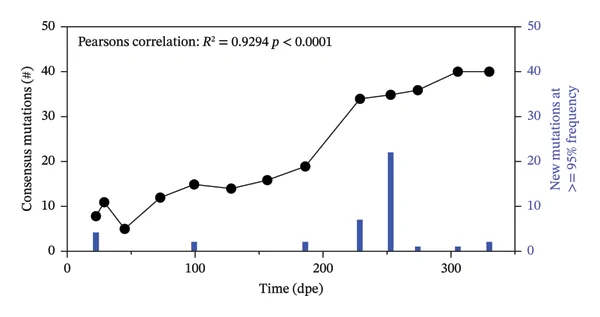
XBK variants. Variants that were at 50% frequency or more, that were not present in the reference baseline isolate of the XBK lineage, increased over time in a largely linear fashion. Total variant burden (black circles and line) and sweep index (blue bars), defined as the number of variants newly crossing ≥ 95% frequency at each time point, are shown over time postenrollment. The variant burden increases gradually and approximately linearly, whereas fixation events occur in discrete bursts. The largest sweep at time point dpe 253 involves simultaneous fixation of multiple variants without a corresponding jump in total variant burden, consistent with clonal or haplotype‐level replacement rather than independent fixation of individual mutations.

With prolonged infection, we expected that SARS‐CoV‐2 would continue accruing within‐host variants. We plotted the number of consensus mutations over time and found a strong positive correlation between days post enrollment (dpe) and total consensus mutation burden (Pearson’s *R* ^2^ = 0.92, *p* < 0.0001). This was consistent with progressive mutation accumulation over time (Figure 3, black circles/line, Table 2). Overlaying mutation burden with a sweep index (Figure 3, blue bars, Table 3), we showed that the number of variants newly reaching near‐fixation (≥ 95% frequency) demonstrated that fixation events occurred episodically rather than continuously (Table 4). While the total variant burden increased gradually, large clusters of variants crossed the fixation threshold simultaneously, most notably at time point d253, with 22 variants reaching ≥ 95% frequency at this time point. Importantly, these fixation events were not accompanied by abrupt increases in total variant count, suggesting that multiple variants swept together as linked sets. This pattern was consistent with episodic clonal or haplotype replacement within the viral population, rather than independent, gradual fixation of individual variants.

**TABLE 3:** Sweep index^a^.

Time point	New ≥ 95%
22	4
29	0
45	0
73	0
99	2
128	0
156	0
186	2
229	7
253	22
274	1
305	1
330	2

^a^To quantify the timing and magnitude of selective sweeps, we defined a sweep index as the number of variants whose population frequency crossed a dominance threshold, in this case 95%, between consecutive time points. Fixation dynamics are dominated by a single major sweep at day 253, during which over 20 variants reach near‐fixation simultaneously.

**TABLE 4:** Pivot table with all variant frequency data^a^.

POS	Mutation	IC‐035‐02_NS	IC‐035‐03_NS	IC‐035‐04_NS	IC‐035‐05_NS	IC‐035‐06_NS	IC‐035‐07_NS	IC‐035‐08_NS	IC‐035‐09_NS	IC‐035‐10_NS	IC‐035‐11_NS	IC‐035‐12_NS	IC‐035‐13_NS	IC‐035‐14_NS
413	orf1ab:p.Thr50Pro										63.64%			
443	orf1ab:p.Val60Ile		8.75%											
520	orf1ab:p.Met85Ile									6.25%			11.11%	
569	orf1ab:p.Glu102Lys			27.72%			6.02%							
670	orf1ab:p.Ser135Arg	99.80%	99.90%	99.93%	99.82%	99.85%	99.82%	99.89%	99.92%	99.85%	100.00%	99.92%	99.95%	
942	orf1ab:p.Arg226Lys									23.33%				
1314	orf1ab:p.Thr350Ile	13.30%												
1427	orf1ab:p.His388Tyr								10.92%					
1457	orf1ab:p.Arg398Cys		25.28%	12.45%	6.61%		8.07%	8.70%	5.67%					
1616	orf1ab:p.Leu451Ile	5.26%	13.13%	7.57%	21.99%	28.13%								
1685	orf1ab:p.Ala474Thr								5.09%					
2276	orf1ab:p.Ile671Val							23.47%						
2744	orf1ab:p.Asp827Tyr													8.44%
2790	orf1ab:p.Thr842Ile	99.69%	99.84%	99.88%	99.89%	99.79%	99.80%	99.84%	99.72%	99.58%	99.78%	99.80%	99.73%	99.75%
2889	orf1ab:p.Val875Ala													6.25%
2912	orf1ab:p.Leu883Met									5.08%				
2936	orf1ab:p.Thr891Ala													6.25%
3042	orf1ab:p.Pro926His	99.80%	99.80%	99.89%	99.87%	99.77%	99.90%	99.41%	99.78%	99.73%	99.63%	99.79%	99.79%	99.86%
3068	orf1ab:p.Asp935Asn								6.76%	18.86%				
3096	orf1ab:p.Ser944Leu		26.80%	13.92%	7.27%		7.84%	9.59%	6.82%					
3117	orf1ab:p.Thr951Ile			17.13%										
3189	orf1ab:p.Gln975Arg													14.29%
3614	orf1ab:p.Val1117Ile								33.90%	59.17%	99.85%	99.78%	99.83%	99.66%
3927	orf1ab:p.Ser1221Leu	99.88%	99.79%	99.72%	99.92%	99.61%	99.80%	99.85%	99.97%	99.94%	99.73%	99.93%	99.85%	99.88%
4184	orf1ab:p.Gly1307Ser	99.97%	99.86%	99.75%	99.75%	99.85%	99.80%	99.85%	99.71%	99.85%	99.93%	99.86%	99.86%	100.00%
4232	orf1ab:p.Asp1323Asn		15.48%		5.00%				33.73%	59.54%	99.16%	99.82%	99.65%	100.00%
4346	orf1ab:p.Ser1361Pro						12.49%							
4379	orf1ab:p.Ser1372Ala								37.16%	53.41%	100.00%	99.74%	99.94%	99.71%
4404	orf1ab:p.Ala1380Val													6.77%
4595	orf1ab:p.Thr1444Ala													33.76%
5030	orf1ab:p.Thr1589Ala		5.65%											
5183	orf1ab:p.Pro1640Ser	99.70%	99.62%	99.81%	99.78%	99.67%	99.73%	99.73%	99.70%	99.65%	99.68%	99.66%	99.62%	99.73%
5547	orf1ab:p.Thr1761Ile						17.43%							
5999	orf1ab:p.Ile1912Val													6.25%
6285	orf1ab:p.Thr2007Ile				5.08%									
6336	orf1ab:p.Ser2024Leu			8.24%			5.95%							
6363	orf1ab:p.Ala2033Val									5.40%				
6402	orf1ab:p.Pro2046Leu											19.91%		
6413	orf1ab:p.Glu2050Lys						26.91%		44.89%	23.84%				
6573	orf1ab:p.Ser2103Phe		27.36%	10.04%			8.17%	9.61%						
6609	orf1ab:p.Arg2115Ile	48.47%	88.09%	38.47%	62.97%	89.73%	42.84%	85.01%	43.12%	19.23%				
6738	orf1ab:p.Thr2158Ile			36.16%			12.37%	5.16%						
6936	orf1ab:p.Ser2224Phe			16.45%										
7142	orf1ab:p.Ser2293Arg					10.28%			6.46%	17.17%	6.04%	16.64%	15.92%	6.27%
7150	orf1ab:p.Leu2295Phe					13.59%			8.96%	21.17%	8.66%	23.34%	21.09%	8.21%
7159	orf1ab:p.Leu2298Phe					14.71%			9.34%	22.44%	9.94%	23.76%	22.42%	7.99%
7167	orf1ab:p.Tyr2301Ser					8.31%				13.42%		12.74%	10.75%	
7177	orf1ab:p.Leu2304Phe					18.50%			12.44%	30.27%	13.28%	28.94%	32.39%	10.22%
7184	orf1ab:p.Ile2307Leu									5.83%		6.20%	5.73%	
7191	orf1ab:p.Ile2309Thr					12.72%			8.09%	19.50%	8.59%	20.63%	18.38%	6.57%
7193	orf1ab:p.Thr2310Pro					7.93%				12.22%	5.33%	11.79%	10.21%	10.30%
7197	orf1ab:p.Ile2311Thr					9.41%			5.68%	15.58%	6.04%	14.57%	12.77%	
7202	orf1ab:p.Ser2313Pro					7.57%			5.47%	6.65%	7.62%	7.09%	5.25%	7.33%
7268	orf1ab:p.Thr2335Pro					7.58%						6.45%		
7281	orf1ab:p.Tyr2339Ser					13.98%			9.81%	10.18%	8.92%	12.62%	6.77%	10.04%
7319	orf1ab:p.Ser2352Arg					5.61%								
7764	orf1ab:p.Ser2500Phe		21.44%	14.70%	5.34%		6.78%	8.43%						
7798	orf1ab:p.Lys2511Asn								31.48%	76.76%	99.81%	99.87%	99.86%	100.00%
8318	orf1ab:p.Pro2685Ser									7.27%				
8354	orf1ab:p.Arg2697Cys					36.84%			26.54%	40.38%	58.98%	49.09%	41.33%	62.90%
8394	orf1ab:p.Ala2710Val								28.50%	79.04%	99.75%	99.74%	99.73%	99.67%
8490	orf1ab:p.Leu2742Ser										8.33%			
8492	orf1ab:p.Thr2743Ala													5.26%
8516	orf1ab:p.Val2751Ile													15.79%
8964	orf1ab:p.Ser2900Leu		9.84%	5.26%	5.40%		7.70%	8.23%						
9042	orf1ab:p.Ser2926Phe	42.26%	30.76%	23.38%	43.34%	35.03%	77.55%	27.50%	99.55%	99.60%	100.00%	99.69%	99.90%	100.00%
9203	orf1ab:p.Asp2980Asn					6.97%								
9344	orf1ab:p.Leu3027Phe	99.78%	99.77%	99.92%	99.90%	99.82%	99.85%	99.73%	99.86%	99.50%	99.86%	99.69%	99.80%	99.83%
9534	orf1ab:p.Thr3090Ile	99.81%	99.78%	99.88%	99.93%	99.82%	99.78%	99.86%	99.74%	99.92%	99.66%	99.71%	99.90%	99.88%
9611	orf1ab:p.Leu3116Phe		18.01%	9.84%										
9711	orf1ab:p.Ser3149Phe	35.20%	8.63%	9.57%	41.08%	11.02%	74.80%	20.14%	94.30%	99.72%	99.74%	99.71%	99.69%	99.53%
9733	orf1ab:p.Phe3156Leu		25.45%	11.63%	5.58%		6.16%	7.00%						
9756	orf1ab:p.Arg3164His	97.25%	74.37%	53.36%	91.79%	95.54%	83.43%	92.41%	94.99%	99.54%	99.83%	99.78%	99.79%	99.66%
9866	orf1ab:p.Leu3201Phe	99.75%	99.83%	99.90%	99.84%	99.67%	99.76%	99.86%	99.85%	99.86%	100.00%	99.94%	99.81%	99.50%
10029	orf1ab:p.Thr3255Ile	99.79%	99.40%	99.66%	99.58%	99.77%	99.75%	99.89%	99.86%	99.73%	99.85%	99.74%	99.75%	99.92%
10152	orf1ab:p.Asp3296Gly				11.76%	18.88%		6.52%						
10286	orf1ab:p.Ile3341Val					5.90%								
10323	orf1ab:p.Lys3353Arg	17.95%												
10376	orf1ab:p.Pro3371Thr				7.14%									
10383	orf1ab:p.Gln3373Leu												8.00%	
10416	orf1ab:p.Ser3384Leu	7.29%	6.79%		15.58%	20.19%								
10449	orf1ab:p.Pro3395His	99.69%	99.75%	99.56%	99.89%	99.78%	99.81%	99.76%	99.82%	99.80%	99.69%	99.83%	99.61%	99.60%
10740	orf1ab:p.Asp3492Val													28.27%
11112	orf1ab:p.Met3616Thr												8.11%	
11216	orf1ab:p.Asn3651His					5.02%								
11296	orf1ab:p.Phe3677Leu	86.96%	76.19%		44.44%	76.43%	9.33%	69.64%	97.72%	14.81%	100.00%	5.36%	69.44%	9.68%
11313	orf1ab:p.Val3683Ala													6.15%
11334	orf1ab:p.Val3690Ala													5.62%
11514	orf1ab:p.Thr3750Ile		29.10%	14.87%	6.81%		9.02%	8.15%						
11623	orf1ab:p.Leu3786Phe					6.52%				9.12%	6.40%	12.98%	9.45%	7.23%
11628	orf1ab:p.Tyr3788Ser											5.23%		
11658	orf1ab:p.Cys3798Phe					9.80%			7.14%	9.16%	5.06%	17.70%	7.45%	11.78%
11669	orf1ab:p.Arg3802Cys									54.22%	99.89%	99.69%	99.73%	100.00%
11697	orf1ab:p.Tyr3811Ser					10.99%			8.58%	8.73%	9.34%	15.02%	8.70%	10.73%
12008	orf1ab:p.Leu3915Phe	36.94%		43.20%	27.98%		67.27%	10.80%	20.17%					
12045	orf1ab:p.Ile3927Thr	35.67%	39.94%	31.97%	27.79%	47.22%	8.57%	74.01%						
12194	orf1ab:p.Leu3977Ile								86.71%	99.94%	99.87%	99.93%	99.88%	100.00%
12402	orf1ab:p.Asn4046Ser													7.01%
12444	orf1ab:p.Asn4060Ser	99.89%	99.93%	99.84%	99.92%	99.90%	99.91%	99.81%	99.92%	99.96%	99.94%	99.91%	99.94%	99.94%
12651	orf1ab:p.Thr4129Ile	41.33%	15.29%	33.76%	33.12%	7.47%	69.97%	11.90%	17.38%					
13034	orf1ab:p.Ala4257Ser											22.12%		
13050	orf1ab:p.Ala4262Val		6.87%			6.69%								
13119	orf1ab:p.Ala4285Val	10.34%												
13128	orf1ab:p.Gly4288Glu		7.15%											
13339	orf1ab:p.Asn4358Lys		23.00%	11.96%			7.00%	8.26%						
13853	orf1ab:p.Asn4530Ser					5.46%								
14277	orf1ab:p.Arg4671Ser													9.62%
14408	orf1ab:p.Pro4715Leu	99.78%	99.27%	100.00%	100.00%	99.57%	99.83%	99.81%	99.51%	99.54%	99.74%	99.80%	99.81%	100.00%
14584	orf1ab:p.Ala4774Thr								79.19%	33.29%				
15181	orf1ab:p.Ala4973Thr								76.01%	44.69%				
15451	orf1ab:p.Gly5063Ser	99.79%	100.00%	99.72%	99.88%	99.82%	99.54%	99.75%	99.81%	99.92%	99.59%	99.38%	99.90%	100.00%
15469	orf1ab:p.Pro5069Thr	5.16%												
15538	orf1ab:p.Val5092Ile								6.63%					
16015	orf1ab:p.Phe5251Ile													5.46%
16311	orf1ab:p.Leu5349Phe					10.58%			8.61%	8.31%	6.22%	7.43%	5.44%	8.62%
16328	orf1ab:p.Tyr5355Ser					20.28%			17.03%	18.32%	18.07%	21.08%	15.00%	20.33%
16348	orf1ab:p.Ser5362Pro					18.15%			14.45%	16.48%	11.81%	16.80%	12.50%	17.27%
16351	orf1ab:p.His5363Asn					12.87%			11.57%	12.54%	13.08%	12.23%	11.51%	12.32%
16408	orf1ab:p.Thr5382Ala		20.81%	10.86%										
16733	orf1ab:p.Ser5490Leu						7.25%	8.75%	7.70%					
16774	orf1ab:p.Tyr5504His													5.38%
16856	orf1ab:p.Asp5531Gly													8.16%
17410	orf1ab:p.Arg5716Cys	99.77%	99.68%	99.75%	99.85%	99.87%	99.92%	99.68%	99.84%	99.94%	99.82%	99.80%	99.76%	99.88%
17746	orf1ab:p.Pro5828Ser										7.76%			
18050	orf1ab:p.Val5929Glu													13.74%
18052	orf1ab:p.Thr5930Ala													9.38%
18086	orf1ab:p.Thr5941Ile											18.16%		
18146	orf1ab:p.Glu5961Gly													9.29%
18163	orf1ab:p.Ile5967Val	99.32%	99.95%	99.73%	99.92%	99.74%	99.95%	99.82%	100.00%	99.84%	100.00%	99.86%	99.76%	100.00%
18181	orf1ab:p.Asp5973Asn						9.43%	6.03%	16.60%					
18499	orf1ab:p.Tyr6079His		22.23%	7.43%										
18583	orf1ab:p.Val6107Ile	99.72%	99.77%	99.83%	99.81%	99.77%	99.85%	99.80%	99.78%	99.81%	99.89%	99.77%	99.74%	99.75%
18862	orf1ab:p.Ile6200Val													5.65%
18947	orf1ab:p.Leu6228Pro								14.73%					
18967	orf1ab:p.Arg6235Gly													5.21%
18985	orf1ab:p.Val6241Ile												53.41%	47.04%
19068	orf1ab:p.Gln6268His						8.15%	5.06%	16.25%					
19378	orf1ab:p.Tyr6372His													19.36%
19955	orf1ab:p.Thr6564Ile	99.84%	99.71%	99.92%	99.77%	99.88%	98.22%	98.91%	99.88%	99.87%	99.83%	99.90%	99.94%	99.94%
19969	orf1ab:p.Ala6569Thr													63.23%
20117	orf1ab:p.Thr6618Ile						7.58%	7.62%						
20132	orf1ab:p.Ala6623Val	47.80%		49.24%	25.74%		47.58%	11.62%	15.84%					
20372	orf1ab:p.Leu6703Gln		21.02%	9.44%										
20392	orf1ab:p.Phe6710Leu						8.55%	9.09%						
20404	orf1ab:p.Pro6714Ser									73.93%	99.70%	99.75%	99.72%	99.76%
20405	orf1ab:p.Pro6714Leu						8.50%	9.03%						
20486	orf1ab:p.Lys6741Arg													22.60%
20762	orf1ab:p.Thr6833Ile						7.01%							
20776	orf1ab:p.Ile6838Val						7.09%	6.95%						
20846	orf1ab:p.Tyr6861Cys						8.09%	8.20%						
20975	orf1ab:p.Asp6904Gly													17.73%
21085	orf1ab:p.Asn6941Asp													6.68%
21137	orf1ab:p.Lys6958Arg	21.93%	30.12%	27.93%	23.91%	23.31%	37.45%	73.09%		74.18%	99.75%	99.75%	99.82%	99.95%
21160	orf1ab:p.Ala6966Thr												11.41%	97.14%
21579	S:p.Val6Ala										13.85%			
21587	S:p.Pro9Ser												58.37%	
21588	S:p.Pro9Leu											5.91%	41.63%	
21593	S:p.Val11Ile												58.37%	
21618	S:p.Thr19Ile	99.85%	100.00%	100.00%	100.00%	100.00%	99.91%	100.00%	99.90%	100.00%	100.00%	100.00%	100.00%	
21641	S:p.Ala27Ser	20.00%												
21641	S:p.Ala27Pro	80.00%												
21668	S:p.Val36Ile				5.15%	15.65%								
21718	S:p.Gln52His	31.10%	56.26%	26.36%	41.33%	76.94%	44.09%	65.24%	75.92%	98.04%	98.95%	99.11%	99.09%	100.00%
21746	S:p.Val62Leu	34.11%	49.34%	34.59%	42.58%	74.25%	8.07%	74.23%		74.25%	100.00%	100.00%	99.85%	99.65%
21752	S:p.Trp64Arg											22.72%		
21754	S:p.Trp64Cys													25.88%
21758	S:p.His66Tyr											5.27%		
21759	S:p.His66Arg											18.29%		
21769	S:p.His69Gln											13.33%		
21770	S:p.Val70Leu											33.33%	40.00%	
21771	S:p.Val70Ala									74.25%	100.00%	99.88%	99.79%	100.00%
21776	S:p.Gly72Arg								7.67%					
21786	S:p.Gly75Asp									19.35%				
21855	S:p.Ser98Phe							7.87%						
21987	S:p.Gly142Asp	99.73%	99.73%	99.19%	99.59%	99.73%	99.82%	99.80%	99.77%	99.72%	99.59%	99.71%	99.79%	99.44%
22001	S:p.Lys147Glu	99.92%	97.85%	99.10%	99.45%	99.71%	99.35%	95.94%	99.85%	99.25%	99.84%	99.80%	99.86%	100.00%
22016	S:p.Trp152Arg	96.21%	87.80%	87.94%	92.76%	97.37%	92.37%	90.45%	97.37%	97.14%	97.03%	96.61%	97.42%	97.53%
22033	S:p.Phe157Leu	99.88%	99.83%	99.81%	99.81%	99.83%	99.91%	99.80%	99.87%	99.37%	99.84%	99.90%	99.93%	99.64%
22190	S:p.Ile210Val	99.96%	99.78%	99.77%	100.00%	99.78%	99.86%	99.87%	100.00%	99.91%	100.00%	99.91%	99.91%	100.00%
22200	S:p.Val213Gly	99.87%	99.73%	99.77%	99.84%	99.93%	99.91%	99.94%	99.86%	100.00%	99.88%	99.88%	99.94%	100.00%
22295	S:p.His245Tyr						10.84%	16.48%	82.77%	99.77%				
22296	S:p.His245Leu											61.54%		
22296	S:p.His245Pro											38.46%		
22329	S:p.Ser256Leu									62.13%	99.84%	99.83%	99.91%	100.00%
22331	S:p.Gly257Ser	99.68%	99.49%	99.78%	99.48%	99.72%	99.71%	99.80%	99.68%	99.83%	99.57%	99.83%	99.82%	100.00%
22352	S:p.Ala264Thr								8.26%					
22413	S:p.Thr284Ile			33.55%			11.82%							
22428	S:p.Val289Ala											10.00%		
22449	S:p.Leu296Pro											10.00%		
22478	S:p.Phe306Val			37.10%			13.46%							
22553	S:p.Asn331Asp													16.20%
22577	S:p.Gly339Arg	100.00%	96.72%	96.70%	100.00%	99.30%	98.56%	98.39%	99.33%	100.00%	98.41%	100.00%	100.00%	100.00%
22578	S:p.Gly339Asp	99.82%	100.00%	99.45%	100.00%	99.91%	99.35%	99.53%	98.89%	99.74%	99.63%	99.84%	99.70%	98.59%
22599	S:p.Arg346Thr	99.54%	100.00%	100.00%	99.48%	99.65%	99.61%	99.53%	99.78%	99.48%	99.88%	100.00%	100.00%	100.00%
22629	S:p.Lys356Thr	82.26%	59.29%	46.14%	87.59%	92.93%	78.45%	93.86%	21.73%	70.59%	100.00%	99.94%	100.00%	100.00%
22674	S:p.Ser371Phe	99.79%	99.85%	99.88%	99.64%	99.82%	99.90%	99.85%	99.70%	99.49%	100.00%	99.81%	99.79%	99.86%
22679	S:p.Ser373Pro	99.79%	99.90%	99.81%	100.00%	99.97%	99.95%	99.93%	99.87%	100.00%	100.00%	99.89%	99.91%	100.00%
22686	S:p.Ser375Phe	99.79%	99.85%	99.81%	99.52%	99.77%	99.84%	99.71%	99.49%	99.86%	99.81%	99.75%	99.76%	98.99%
22688	S:p.Thr376Ala	99.73%	99.65%	99.81%	99.14%	99.90%	99.49%	99.75%	99.70%	99.93%	100.00%	99.94%	99.69%	100.00%
22770	S:p.Arg403Lys								23.14%	7.79%				
22771	S:p.Arg403Ser							31.84%						
22775	S:p.Asp405Asn	45.03%	79.00%	42.22%	62.26%	75.37%	20.69%	79.97%	72.89%	81.45%	79.43%	80.39%		
22786	S:p.Arg408Ser	99.78%	99.89%	99.78%	99.51%	99.83%	99.87%	99.79%	99.86%	99.92%	99.92%	99.86%	99.83%	99.85%
22813	S:p.Lys417Asn	99.96%	99.93%	100.00%	100.00%	99.78%	99.90%	99.84%	99.89%	99.77%	99.93%	99.90%	99.94%	99.63%
22881	S:p.Asn440Ser								19.97%	7.67%				
22882	S:p.Asn440Lys	99.93%	99.95%	99.96%	99.87%	99.98%	99.93%	99.90%	99.65%	99.96%	99.90%	99.98%	99.95%	100.00%
22893	S:p.Lys444Met	5.31%												
22893	S:p.Lys444Thr	38.35%	62.07%	40.28%	58.33%	85.50%	9.99%	78.36%		77.56%	99.93%	99.86%	99.86%	100.00%
22898	S:p.Gly446Ser	99.85%	99.77%	99.76%	99.76%	99.75%	99.86%	98.22%	79.87%	92.29%	99.83%	99.85%	99.82%	99.89%
22925	S:p.Leu455Met	49.10%		41.99%	29.77%	6.34%	71.61%	12.05%	22.94%					
22942	S:p.Asn460Lys	83.00%	96.83%	85.01%	92.85%	86.75%	85.68%	94.48%	79.81%	88.20%	85.78%	91.84%	88.77%	90.10%
22971	S:p.Thr470Asn	6.30%												
22988	S:p.Gly476Ser								22.96%	27.57%				
22992	S:p.Ser477Asn	98.99%	99.02%	98.83%	99.45%	96.65%	99.10%	98.97%	97.23%	99.12%	99.72%	99.54%	99.64%	99.47%
22993	S:p.Ser477Arg									71.95%	99.49%	99.51%	99.62%	99.34%
22995	S:p.Thr478Lys	98.97%	99.22%	98.83%	99.41%	97.29%	99.24%	98.92%	97.83%	98.99%	99.49%	99.57%	99.55%	99.74%
23013	S:p.Glu484Ala	99.92%	99.80%	99.88%	99.91%	99.94%	99.94%	99.89%	99.96%	99.93%	99.88%	99.92%	99.55%	49.54%
23013	S:p.Glu484Val													50.46%
23014	S:p.Glu484Asp								56.13%	7.48%				
23018	S:p.Phe486Leu	99.92%	99.90%	99.88%	99.96%	99.80%	99.91%	99.90%	99.83%	99.89%	99.82%	99.88%	99.90%	99.90%
23019	S:p.Phe486Ser	99.95%	99.88%	99.74%	99.87%	99.91%	99.88%	99.84%	99.93%	99.96%	99.97%	99.95%	99.97%	97.53%
23031	S:p.Phe490Ser	99.92%	99.74%	99.88%	100.00%	99.92%	99.94%	99.86%	99.86%	99.99%	99.85%	99.66%	99.80%	96.10%
23041	S:p.Gln493His													46.02%
23048	S:p.Gly496Ser													45.81%
23055	S:p.Gln498Arg	99.84%	99.94%	99.80%	99.89%	99.77%	99.83%	99.79%	99.81%	99.84%	99.68%	99.78%	99.86%	99.90%
23063	S:p.Asn501Tyr	99.80%	99.88%	100.00%	99.87%	99.82%	99.86%	99.89%	99.92%	99.83%	99.77%	99.86%	99.82%	100.00%
23069	S:p.Val503Leu	38.25%	62.85%	40.37%	58.14%	85.92%	10.09%	79.86%		88.86%	99.55%	99.91%	99.88%	100.00%
23075	S:p.Tyr505His	99.85%	99.92%	99.88%	99.89%	99.94%	99.92%	99.90%	99.92%	99.80%				
23099	S:p.Leu513Phe	7.44%	15.57%		5.92%	5.79%	5.32%		71.61%					
23108	S:p.Glu516Gln	77.86%	47.77%	82.27%	85.00%	83.40%	91.25%	73.18%	23.47%					
23277	S:p.Thr572Ile							8.63%		67.06%	99.20%	99.74%	99.87%	99.85%
23403	S:p.Asp614Gly	99.93%	99.89%	99.77%	99.90%	99.90%	99.83%	99.83%	99.91%	99.90%	99.71%	99.80%	99.91%	100.00%
23423	S:p.Pro621Ser			6.77%				45.66%						
23447	S:p.Leu629Val													41.44%
23453	S:p.Pro631Ser	35.25%		5.70%	30.09%	6.23%	62.68%	13.54%	22.81%					
23474	S:p.Thr638Pro					8.88%			6.26%	7.42%	5.73%	11.15%	5.76%	7.68%
23520	S:p.Ala653Val		28.53%	12.20%	6.27%		8.07%	9.11%	6.20%					
23525	S:p.His655Tyr	99.92%	99.98%	100.00%	99.92%	99.82%	99.81%	99.94%	99.84%	99.79%	99.84%	99.83%	99.80%	99.67%
23595	S:p.Thr678Ile						5.95%		49.62%	12.12%				
23599	S:p.Asn679Lys	100.00%	100.00%	100.00%	99.85%	99.90%	100.00%	99.73%	24.09%	6.78%	97.22%	99.94%	99.87%	100.00%
23604	S:p.Pro681His	99.88%	100.00%	99.87%	100.00%	99.75%	99.46%	98.90%	99.80%	99.84%	99.79%	99.63%	99.63%	100.00%
23624	S:p.Ala688Ser								5.28%	11.96%				
23625	S:p.Ala688Val							27.47%		84.30%	99.90%	99.83%	99.85%	100.00%
23673	S:p.Ser704Leu						10.47%	15.59%	32.18%	10.65%				
23854	S:p.Asn764Lys	79.89%	69.97%	64.12%	78.90%	81.98%	70.08%	73.09%	81.00%	76.80%	82.86%	76.73%	87.71%	71.76%
23856	S:p.Arg765His		10.29%											
23948	S:p.Asp796Tyr	97.47%	99.17%	94.25%	99.56%	97.15%	95.48%	55.55%	99.88%	99.86%	99.78%	99.86%	99.89%	100.00%
23948	S:p.Asp796His			5.62%				44.42%						
23954	S:p.Gly798Ser											39.13%	56.33%	9.00%
24062	S:p.Ile834Val						8.27%							
24083	S:p.Leu841Phe													12.37%
24089	S:p.Asp843Asn	18.13%		43.57%			12.20%							
24090	S:p.Asp843Gly			9.41%			9.31%	50.36%						
24365	S:p.Gln935Glu		28.50%	11.31%	5.41%		7.69%	8.74%	7.28%					
24371	S:p.Ser937Ala							27.58%						
24398	S:p.Gly946Arg			5.55%	9.69%	22.64%		10.07%						
24420	S:p.Asn953Ser		26.73%	11.65%	6.01%		7.24%	8.47%	7.90%					
24423	S:p.Gln954Arg	43.37%		40.32%	33.89%	7.16%	79.16%	15.03%	25.86%					
24424	S:p.Gln954His	99.86%	99.85%	99.92%	99.67%	99.81%	99.87%	99.86%	99.85%	99.85%	99.95%	99.78%	99.83%	
24441	S:p.Asn960Thr	47.29%	75.70%	41.23%	61.76%	88.80%	12.50%	72.39%	66.98%	99.83%	100.00%	99.94%	99.95%	
24441	S:p.Asn960Ser							6.32%						
24453	S:p.Lys964Ile											57.26%	99.95%	
24463	S:p.Ser967Arg												42.80%	
24468	S:p.Asn969Ser							6.35%						
24469	S:p.Asn969Lys	99.69%	99.80%	99.84%	99.90%	99.75%	99.88%	99.63%	99.84%	99.56%	99.95%	99.83%	99.92%	
24495	S:p.Asn978Ser	10.90%	24.51%	13.02%	6.43%		7.82%	7.74%	7.46%					
24497	S:p.Asp979Tyr											7.07%		
24532	S:p.Glu990Asp						18.18%							
24863	S:p.His1101Tyr									67.54%	99.96%	99.86%	99.86%	100.00%
24927	S:p.Val1122Ala													5.56%
24929	S:p.Ser1123Pro													11.11%
24938	S:p.Cys1126Ser													5.56%
24938	S:p.Cys1126Arg													5.56%
24939	S:p.Cys1126Tyr		11.50%											
24945	S:p.Val1128Ala													5.56%
25010	S:p.Glu1150Lys											18.42%		
25012	S:p.Glu1150Asp													43.33%
25019	S:p.Asp1153Tyr											35.22%	55.82%	56.55%
25019	S:p.Asp1153His												13.03%	
25046	S:p.Pro1162Ser		8.45%	5.46%			11.24%	9.55%	5.58%					
25047	S:p.Pro1162Leu	10.50%					7.06%	8.12%						
25484	ORF3a:p.Ala31Asp							5.70%						
25496	ORF3a:p.Ile35Thr						5.13%		72.39%	30.56%				
25549	ORF3a:p.Leu53Phe									67.14%	99.66%	99.60%	99.67%	100.00%
25552	ORF3a:p.Ala54Ser									67.27%	99.95%	99.81%	99.80%	100.00%
25639	ORF3a:p.Leu83Val	5.35%												
25784	ORF3a:p.Trp131Leu			12.18%			11.84%							
25892	ORF3a:p.Ile167Thr													5.27%
26060	ORF3a:p.Thr223Ile	99.81%	99.52%	100.00%	99.58%	99.83%	99.87%	100.00%	99.91%	99.84%	100.00%	99.93%	99.70%	100.00%
26270	E:p.Thr9Ile	100.00%	100.00%	99.73%	100.00%	99.86%	100.00%	100.00%	99.71%	99.85%	99.85%	99.86%	99.81%	99.38%
26275	E:p.Thr11Ala	99.67%	99.76%	99.73%	99.73%	99.86%	100.00%	100.00%	99.41%	99.85%	100.00%	99.95%	100.00%	100.00%
26416	E:p.Val58Leu								68.51%	31.58%				
26425	E:p.Arg61Cys		26.02%	13.47%	6.45%		7.58%	8.17%	5.79%					
26530	M:p.Asp3Gly													52.14%
26533	M:p.Ser4Phe	82.51%	62.50%	79.56%	88.91%	92.35%	87.24%	76.13%	25.61%	69.28%	99.82%	99.92%	99.56%	99.75%
26538	M:p.Gly6Ser								69.30%	30.72%				
26539	M:p.Gly6Asp	9.84%												
26550	M:p.Val10Ile		25.65%	11.14%			7.83%	7.98%	5.40%					
26577	M:p.Gln19Glu	99.29%	94.74%	95.65%	100.00%	99.55%	96.63%	97.59%	99.56%	100.00%	100.00%	98.40%	99.41%	
26620	M:p.Cys33Tyr		5.56%											
26709	M:p.Ala63Thr	99.10%	100.00%	100.00%	99.53%	99.59%	99.85%	99.91%	100.00%	100.00%	99.26%	99.88%	99.70%	100.00%
26728	M:p.Ala69Val	8.48%						13.10%						
26895	M:p.His125Tyr								7.37%					
27382	ORF6:p.Asp61His	99.96%	100.00%	99.77%	100.00%	99.79%	99.70%	99.87%	99.98%	99.92%	99.96%	99.78%	99.90%	99.71%
27383	ORF6:p.Asp61Val	99.69%	99.69%	99.54%	99.51%	99.74%	99.63%	99.40%	99.75%	99.72%	99.64%	99.52%	99.70%	99.86%
27632	ORF7a:p.Arg80Ile													58.62%
27679	ORF7a:p.Leu96Ile								41.61%	33.31%				
27755	ORF7a:p.Glu121Gly													6.55%
27904	ORF8:p.Leu4Pro												7.69%	
27933	ORF8:p.Ala14Thr	41.85%		6.68%	9.38%							5.00%		
27939	ORF8:p.Phe16Leu											10.00%		
28006	ORF8:p.Pro38Leu	41.87%		6.82%	26.93%		26.25%	11.48%	14.81%					
28254	ORF8:p.Ile121Phe		8.21%											
28254	ORF8:p.Ile121Leu		17.50%											
28311	N:p.Pro13Leu	99.73%	99.84%	99.77%	99.80%	99.73%	99.57%	99.60%	99.57%	99.78%	99.77%	99.75%	99.76%	99.60%
28365	N:p.Glu31Val					84.62%								
28367	N:p.Arg32Cys											95.24%		
28368	N:p.Arg32Pro											8.70%		
28370	N:p.Ser33Gly	37.80%	30.77%	47.06%	60.87%	58.33%	42.25%	40.54%	69.12%	66.67%	50.00%	76.30%	61.17%	78.95%
28370	N:p.Ser33Cys	31.71%	30.77%	23.53%	13.04%	5.77%	15.49%	17.12%						
28370	N:p.Ser33Arg			5.88%										
28382	N:p.Ser37Pro		8.34%	6.18%	7.17%		19.96%	11.58%						
28395	N:p.Arg41Gln													5.17%
28419	N:p.Thr49Ile			6.10%			5.11%							
28610	N:p.Leu113Ile							8.58%	90.91%	99.80%	99.81%	99.80%	99.77%	99.95%
28821	N:p.Ser183Phe													100.00%
28881	N:p.Arg203Lys	99.87%	99.80%	99.83%	99.87%	99.68%	99.80%	99.86%	99.82%	99.26%	99.56%	99.69%	99.63%	99.48%
28883	N:p.Gly204Arg	99.93%	99.94%	99.94%	99.96%	99.92%	99.93%	99.93%	99.88%	99.88%	99.96%	99.81%	99.92%	100.00%
28907	N:p.Gly212Cys			7.01%			16.95%							
28913	N:p.Gly214Cys						12.48%							
28975	N:p.Met234Ile						19.44%							
29003	N:p.Gln244Lys			5.95%			6.97%	25.62%						
29320	N:p.Gln349His									12.96%				
29409	N:p.Thr379Ile			7.54%			5.11%							
29433	N:p.Lys387Arg	8.35%	12.54%			26.30%								
29434	N:p.Lys387Asn		29.56%	12.76%	7.69%	5.10%	9.45%	15.98%	6.03%					
29510	N:p.Ser413Arg	99.75%	99.95%	99.83%	99.72%	99.97%	99.63%	99.87%	99.89%	99.99%	99.98%	99.89%	99.97%	99.91%
29518	N:p.Asp415Glu									8.10%				
29627	ORF10:p.Arg24Cys		40.15%	14.57%	8.74%	5.42%	13.68%	11.88%	8.14%					
29663	ORF10:p.Asn36Tyr	15.86%		16.08%			11.99%		41.22%	82.46%	99.84%	99.71%	99.84%	99.73%

^a^At some time points, our script outputted two different variants, both at > 90% frequency. For these cases, the variants listed are incorrect because the actual variant present is due to a codon that has two changes and results in a totally different amino acid residue than either of those listed. This occurred three times: ORF6:D61L, S:G339H, and S:F486P.

Cryptic lineages have been hypothesized to emerge in IC patients with prolonged viral replication [10–12]. Consistent with this, we observed the emergence of two mutations often seen in cryptic lineages, S:K444T and S:T572I at d229, becoming fixed at d253.

## 3. Discussion

In this case report, the patient tested positive for SARS‐CoV‐2 in either nasal swabs or stool for almost a year and reported experiencing COVID‐like symptoms for several months prior to enrollment. Based on the XBK variant we sequenced, and the time frame when that variant was most prevalent in the community, it is likely that this IC participant had been infected for at least several months before enrolling in the study. Although new variants arose throughout the observation period, fixation occurred episodically, with large clusters of mutations swept to near‐fixation simultaneously. This pattern is consistent with clonal (haplotype‐level) replacement rather than independent, gradual fixation of individual mutations. These new mutations included two that are often seen in cryptic lineages.

Persistent SARS‐CoV‐2 infections endanger the individual and, more broadly, the population by sustaining the potential for transmission to others. According to the CDC, it is estimated that from October 2024 to September 2025, there have been between 13.8 and 20.3 million infections of SARS‐CoV‐2 and 44,000‐63,000 deaths due to SARS‐CoV‐2 in the United States [13]. In the general population, 6% of SARS‐CoV‐2 infections have been shown to have viral shedding after 30 days since onset, and it is estimated that between 0.1% and 0.5% of individuals who contract SARS‐CoV‐2 infection become persistently infected [14]. Swank et al. found that 40% of individuals with long COVID symptoms in the postacute phase tested antigen‐positive and 20% of all individuals had persistent antigen production between 1‐month and 14‐month postinfection [15].

The true prevalence of SARS‐CoV‐2 infections, viral shedding, and persistent cases in the population remains uncertain. However, IC individuals are at an increased risk for severe and prolonged SARS‐CoV‐2 infection due to impaired immune‐mediated viral clearance [3–5]. In this population, COVID‐19 may follow a persistent or relapsing course with extended viral shedding and increased morbidity. Population‐level data from the INFORM study demonstrate that IC patients continue to account for a disproportionate share of COVID‐19‐related hospitalizations and deaths, underscoring their ongoing vulnerability [16].

The viral mutations noted in persistent SARS‐CoV‐2 infections in IC individuals are relevant for treatment and management of SARS‐CoV‐2. Our patient exhibited two viral mutations with changes often found in cryptic lineages. Leveraging novel cellular therapies may help to curtail the length of infections and mutations seen in these cases. Direct‐acting antiviral treatment has also shown promise in the treatment of persistent SARS‐CoV‐2 in mouse models, but clinical trials are needed to better understand patient outcomes [17]. While people experiencing long COVID may utilize longer duration Paxlovid [18, 19], this is difficult in a person on transplant immunosuppression because ritonavir is a very strong CYP3A4 inhibitor and calcineurin inhibitors are metabolized by CYP3A4, potentially resulting in toxic blood levels of these medications [20–22]. High titer convalescent plasma can also be an adaptive treatment option for persistent SARS‐CoV‐2 infections in IC individuals as it can be collected locally, matching circulating variants and rarely produces adverse effects [23, 24].

Our patient received several SARS‐CoV‐2‐directed therapies before enrollment in our study. Two years before enrollment, after testing positive for COVID‐19, the patient received a single intravenous infusion of casirivimab/imdevimab (REGEN‐COV). The following year, the patient was given tixagevimab (300 mg) and cilgavimab (300 mg) (Evusheld) as a one‐time injection for pre‐exposure prophylaxis.

In March 2023, the patient was treated with remdesivir for 4 days in combination with dexamethasone (6 mg daily for 10 days), a regimen shown to be effective against the Omicron variant and which remains FDA‐approved for the treatment of COVID‐19 [13, 14]. However, while remdesivir plus dexamethasone is effective as antiviral therapy, it does not provide the same duration of protection as monoclonal antibody therapy.

As of 2025, neither casirivimab/imdevimab nor tixagevimab/cilgavimab is used for SARS‐CoV‐2 treatment due to reduced activity against currently circulating variants. Nine months after the March 2023 treatment, the patient was enrolled in our study.

## 4. Conclusion

Persistent SARS‐CoV‐2 infections in IC individuals continue to be an area of concern for the development of SARS‐CoV‐2 variants. This case report of an IC patient living with a B‐cell deficiency who tested positive for SARS‐CoV‐2 for nearly a year highlights the time‐dependent nature of SARS‐CoV‐2 viral mutation and the potential for new cryptic lineages to arise from long‐term infections. Continued efforts to develop antiviral therapies that can provide adequate protection from SARS‐CoV‐2 infection persistence could improve outcomes for IC patients and slow the development of new lineages over time.

## Author Contributions

All authors contributed to the conception, design, drafting, and revision of the manuscript.

## Funding

Initial funding was provided by impact of immune failure on SARS‐CoV‐2 evolutionary potential, CDC Contract Number: 200‐2021‐11060‐ FY21 BAA Topic 8 Wisconsin‐Madison (David H. O’Connor and Thomas C. Friedrich are co‐principal investigators). Listed currently on JGW’s institutional review board. DHO/TCF (previously Immune Failure) Discretionary Funds. DHO/TCF 435100‐A24‐ELCProjE‐00 Wisconsin Department of Health Services, ELC Project: E − Enhancing Detection Expansion. Final funding was provided by Heart of Racing GF000020542 DHO. JGW Departmental Start‐Up Funds.

## Conflicts of Interest

The authors declare no conflicts of interest.

## Supporting Information

Additional supporting information can be found online in the Supporting Information section.

## Supporting information


**Supporting Information 1** Supporting Table 1. Similarity analysis. This was a similarity analysis between IC‐035‐02 and XBK sequences uploaded to GenBank that were nearer in time and distance to the patient than the baseline reference sequence we chose. We downloaded a listing of all of the XBK sequences that had been uploaded to GenBank. The earliest, OY837126, was chosen as the baseline lineage upon which we based our analysis of consensus mutation changes over time. We then filtered the list by samples taken in the USA and looked for the 19 most recent samples that were not samples we uploaded. We input these 19, along with OY837126 and IC‐035‐02, into Nextclade using the Wuhan strain as the comparator sequence. The output nextclade.tsv file was analyzed in python (Compare_XBK.ipynb—in the data portal); the output is a similarity analysis in which each sequence is compared to IC‐035‐02 and then ranked by the number and kind of mutations. Mutations_NOT_in_Ref are mutations present in the comparator sequences that are not present in IC‐035‐02 but are present in those sequences. Mutation_Missing_from_sample are mutations present in IC‐035‐02 but not in the comparator sequence. The baseline reference sequence is highlighted in orange and is in the middle of all the sequences expected to be most like 02‐NS. Therefore, we do not feel that our choice of this sequence as the baseline reference is biased.

**Supporting Information 2** CARE‐checklist‐English‐2013 completed.

## Data Availability

The data that support the findings of this study are openly available in Persistent SARS‐CoV‐2 Infection Outcomes and Mutations Associated with B‐Cell Deficiency at https://dholk.primate.wisc.edu/dho/public/manuscripts/published/Persistent%20SARS-CoV-2%20Infection%20Outcomes%20and%20Mutations%20Associated%20with%20B-Cell%20Deficiency/project-begin.view?wizard=true.
