# Supplementary material for: Describing a Persistent SARS‐CoV‐2 Infection Outcomes and Mutations Associated With B‐Cell Deficiency: A Case Report
Source: Case Rep Infect Dis. 2026 Sep 24;2026:6154094. doi: 10.1155/crdi/6154094 (PMC13602350; doi:10.1155/crdi/6154094)
Supplement: Supplementary file 1 [file CRDI-2026-6154094-s002.pdf]

CARE Checklist of information to include when writing a case report

CARE case report guidelines

(cc) BY-NC-ND

Topic	Item	Checklist item description	Reported on Line
Title	1	The diagnosis or intervention of primary focus followed by the words "case report"	1-2
Key Words	2	2 to 5 key words that identify diagnoses or interventions in this case report, including "case report"	62-63
Abstract (no references)	3a	Introduction: What is unique about this case and what does it add to the scientific literature?	42-45
	3b	Main symptoms and/or important clinical findings	44; 48-55
	3c	The main diagnoses, therapeutic interventions, and outcomes	48-55
	3d	Conclusion—What is the main "take-away" lesson(s) from this case?	57-60
Introduction	4	One or two paragraphs summarizing why this case is unique (**may include references**)	83-96
Patient Information	5a	De-identified patient specific information.	101-109
	5b	Primary concerns and symptoms of the patient.	101-103; 186-188
	5c	Medical, family, and psycho-social history including relevant genetic information	103-106
	5d	Relevant past interventions with outcomes	103-106;228-240
Clinical Findings	6	Describe significant physical examination (PE) and important clinical findings.	112-128; 146-184
Timeline	7	Historical and current information from this episode of care organized as a timeline	101-109;138-144
Diagnostic Assessment	8a	Diagnostic testing (such as PE, laboratory testing, imaging, surveys).	112-145; 147-184
	8b	Diagnostic challenges (such as access to testing, financial, or cultural)	140-144
	8c	Diagnosis (including other diagnoses considered)	102; 146-166
	8d	Prognosis (such as staging in oncology) where applicable	141-143
Therapeutic Intervention	9a	Types of therapeutic intervention (such as pharmacologic, surgical, preventive, self-care)	228-237
	9b	Administration of therapeutic intervention (such as dosage, strength, duration)	228-237
	9c	Changes in therapeutic intervention (with rationale)	N/A observational study
Follow-up and Outcomes	10a	Clinician and patient-assessed outcomes (if available)	138-144; 186-194
	10b	Important follow-up diagnostic and other test results	131-185
	10c	Intervention adherence and tolerability (How was this assessed?)	N/A observational study
	10d	Adverse and unanticipated events	N/A
Discussion	11a	A scientific discussion of the strengths AND limitations associated with this case report	143-144; 186-194
	11b	Discussion of the relevant medical literature **with references**.	196-240
	11c	The scientific rationale for any conclusions (including assessment of possible causes)	186-194; 241-248
	11d	The primary "take-away" lessons of this case report (without references) in a one paragraph conclusion	241-248
Patient Perspective	12	The patient should share their perspective in one to two paragraphs on the treatment(s) they received	N/A observational study
Informed Consent	13	Did the patient give informed consent? Please provide if requested	Yes ☒ No ☐
